# Building health system resilience in the context of primary health care revitalization for attainment of UHC: proceedings from the Fifth Health Sector Directors’ Policy and Planning Meeting for the WHO African Region

**DOI:** 10.1186/s12919-020-00203-2

**Published:** 2020-12-03

**Authors:** Prosper Tumusiime, Humphrey Karamagi, Regina Titi-Ofei, Michelle Amri, Aminata Binetou Wahebine Seydi, Hillary Kipruto, Benson Droti, Sosthene Zombre, Zabulon Yoti, Felicitas Zawaira, Joseph Cabore

**Affiliations:** 1 WHO Regional Office for Africa, PB 06 Cité du Djoue, Brazzaville, Congo; 2 Dalla Lana School of Public Health, University of Toronto, 155 College St, Toronto, Ontario M5T 1P8 Canada; 3 Takemi Program in International Health, Harvard School of Public Health, Harvard University, 665 Huntington Avenue, Bldg. 1, Room 1210, Boston, MA 02115-6021 USA

**Keywords:** Primary health care, Health systems, Global health, Health governance, Multisectoral action, Public policy, Health policy, Universal health coverage, Primary health care, Sustainable development Goals

## Abstract

**Background:**

The recent 2018 Declaration of Astana recognized primary health care (PHC) as a means to achieve universal health coverage (UHC) and the health-related Sustainable Development Goals (SDGs). Following this declaration, country progress on operationalization of the PHC agenda and attainment of UHC has been stalled by the new challenges posed by the COVID-19 pandemic. The pandemic has also disrupted the continuity of essential health service provision and tested the resilience of the region’s health systems.

**Methods:**

In accordance with this, the WHO Regional Office for Africa convened the Fifth Health Sector Directors’ Planning and Policy Meeting across the 47 Member States of the Region. The two-day forum focused on building health system *resilience* to facilitate service continuity during health threats, PHC revitalization, and health systems strengthening towards UHC.

**Results:**

The Regional Forum provided evidence on building resilient health systems in the WHO African Region and engaged participants in meaningful and critical discussion. It is from these discussions that four key themes emerged: (1) working multisectorally/intersectorally, (2) moving from fragmentation to integration, (3) ensuring implementation and knowledge exchange, and (4) rethinking resilience and embracing antifragility. These discussions and associated groupings by thematic areas lend themselves to recommendations for the WHO.

**Conclusions:**

This paper details the proceedings and key findings on building resilient health systems, the four themes that emerged from participant deliberation, and the recommendations that have emerged from the meeting. Deliberations from the Regional Forum are critical, as they have the potential to directly inform policy and program design, given that the meeting convenes health sector technocrats, who are at the helm of policy design, action, and implementation.

## Background

Dr. Tedros Adhanom Ghebreyesus, Director-General of WHO, has emphasized his top priority at the World Health Organization (WHO) is Universal Health Coverage (UHC) [1]. UHC entails “all people have access to the health services they need, when and where they need them, without financial hardship” [2]. In terms of which health services are embedded in UHC, it encompasses more than just treatment, but rather a full range of essential health services, including: health promotion, prevention, rehabilitation, and palliative care [2]. This consideration of health in a holistic manner, as per the definition of health in the WHO constitution [3], is also reflected in the aims of primary health care (PHC), where the concept moved from a traditionally biomedical approach to one focused on prevention, well-being, addressing health inequities, empowering communities, and others [4]. As such, in striving to achieve UHC, the recent 2018 Declaration of Astana recognized PHC as a means to achieve UHC and the health-related Sustainable Development Goals (SDGs) [4]. But it is worth noting, the role of PHC has been recognized as an important aspect of global health for many years, with perhaps the most noteworthy emergence through the 1978 Declaration of Alma-Ata. The understanding that focusing on UHC and PHC are not new but are of particular importance at present, given the COVID-19 pandemic, is paramount. This pandemic has strengthened the case of strengthening the interlinkages between aspirations of UHC and health security— two agendas which should be thought of as being two sides of one coin. This demonstrates the need for strong and resilient health systems, the vehicle through which countries can best prepare for, respond, and recover from the negative impacts of health emergencies [5].

Against this background, the Regional Forum on Health System Strengthening for UHC and SDGs, which serves as an annual assembly of the top-level technical decision makers from the 47 member states comprising the WHO African Region, was convened to deliberate on emerging issues relating to health systems development in the context of UHC and other health-related SDG targets, and prioritize these discussions. The meeting was first convened in Namibia in 2016, where the Regional Framework for Health Systems Strengthening for UHC and SDGs was deliberated on and eventually adopted by the Ministers of Health at the 67th Regional Committee for Africa meeting in 2017 [6, 7]. Each subsequent forum has focused on specific aspects relating to health systems development.

This fifth forum took place in a changing context of health. The Member States have all faced significant and unanticipated challenge to their capacities through the COVID-19 pandemic. The COVID-19 pandemic has changed the context of health and impacted health outcomes globally, emphasizing the crucial role PHC plays [4]. With COVID-19 testing health systems, this Regional Forum focused on building health system *resilience* to facilitate service continuity during health threats, PHC revitalization, and health systems strengthening towards UHC. Together with this, the Astana Declaration on Primary Health Care and the adoption of the SDG 3 Global Action Plan all call for a re-think of the design, focus, and monitoring of health systems development in the WHO Region for Africa.

The central theme of *resilience* was thus timely, as it provided a platform for deliberation on how country health system development needs to be re-aligned with current needs, to ensure suitability in rapidly changing contexts. Sustaining health systems during shocks, such as the COVID-19 pandemic and rebuilding the resultant fragile systems it leaves in its wake, were highlighted as key priorities for countries, as they work to *build back better*. Investing in health system strengthening builds *resilience*, which may bridge the overlapping concepts within competing global health security and UHC approaches [5] (i.e. addressing both collective and independent security respectively [8]). Accordingly, the Regional Forum afforded discussion on: the application of health system resilience to facilitate service continuity and health security; the revitalization of PHC (particularly in relation to a comprehensive PHC approach for UHC attainment and health security); and reviewing key principles in sustaining the provision of essential health services during shocks, and key areas of WHO support to capacitate countries, in this regard. These discussions are under direct alignment with the triple billion strategic priorities of WHO’s 13th General Programme of Work (2019–2023): achieving UHC, addressing health emergencies, and promoting healthier populations [9].

Across the deliberations, four key themes emerged: (1) working multisectorally/intersectorally, (2) moving from fragmentation to integration, (3) ensuring implementation and knowledge exchange, and (4) rethinking resilience and embracing antifragility. These discussions and associated groupings by thematic areas lend themselves to recommendations for the WHO. This manuscript details key findings on building resilient health systems, the four themes that emerged from participant deliberation, and the recommendations that have emerged from the meeting. Deliberations hosted at the Regional Forum are vitally important, as they have the potential to directly inform policy and program design, given that the meeting convenes health sector directors. For example, deliberations and agreements from countries will aim to provide recommendations to contribute to ensuring continuity of essential health services during crises and to implement the framework for action of the Astana Declaration on PHC.

## Methods

The forum was organized around three key sessions on health system resilience, PHC operationalization and Continuity of Essential Health Services in the context of health emergencies. Each session comprised of an introductory presentation of the concept and opportunities for roll out at country level. It was then followed by a group discussion based on specific predetermined questions and then an open-ended conversation in plenary, involving deliberations on emerging issues and lessons learned from the presentations.

### Participants

The meeting convened over 200 participants from the 47 Countries of the WHO African Region, which includes: in-country directors of policy and planning, directors of disease control, directors of monitoring and evaluation, and WHO Country Office health systems and disease control officers; key WHO Regional Office for Africa participants; key directors and technical staff from WHO Headquarters; development partners working within the WHO African Region at the regional and the country levels (invited by the WHO Representatives or their regional/global offices); and Health Bureaus of the various Regional Economic Committees.

## Results

### Building resilient health systems

Disease shock events are still multiple and affect all countries. Ninety-six events were documented (i.e. hit the threshold and followed-up on) in 2018 alone [10]. As a result, countries remain vulnerable, even with the significant investments made in enhancing emergency preparedness and response – and in fact, some shock events will overwhelm any system, such as COVID-19. There are two types of shock events: acute events (short duration), such as Ebola Virus Disease and COVID-19, and also chronic events (longer duration/repetitive), such as cholera outbreaks and measles [11]. Both types have associated environmental, economic, and political events. As a result, the need for different capacities emerges for a resilient system, which is “the capacity to sustain essential services provision, even in the context of shock events” [11, 12].

In looking over the evidence and experiences in the region, including those shared at the fourth Regional Forum, lessons can be drawn from shock events. In such cases, shock events increase service needs, beyond the capacity of the system. Systems that are not resilient will be unable to respond. There are three approaches to avoid the impact of the shock event to ensure services provided by the system are within-capacity: (1) increase inherent capacity of existing system to provide services above its capacity (maximize resilience dividend), (2) increase capacity to force down the shock event (minimize shock event, by building emergency preparedness and response capacity), and (3) increase overall capacity of the system (raise event horizon). Resilience entails working across these three areas – ignoring one will limit the ability to anticipate, absorb, and adapt when facing shock events. These three capacities are needed for a resilient health system.

First, inherent resilience, which was presented as the inherent and inbuilt characteristics or design of the system that enable it to absorb shocks, or succumb to them. Adapted from literature, there are five capacities: awareness capacity, diversity capacity, mobilization capacity, self-regulation capacity, and transformation capacity [12]. These five capacities define a system’s inherent resilience (i.e. built into the system). Analyzing the inherent resilience capacities of the countries in the African Region [13], there is more work needed. Evidently, the largest challenge is the limited adaption from past experiences, as some systems face the same issues over time. While many lessons learned emerged from fighting the 2014–2016 Ebola Virus Disease outbreak, the question remains: how well have those lessons been utilized to adapt our inherent resilience? There are many examples of resilience strengthening interventions in the health system, as detailed in Table 1, which a country may decide to implement.

**Table 1:** Examples of Resilience Strengthening Interventions

Investment area	Resilience strengthening interventions
**Health workforce**	▪ Training on surveillance, risk communication, partner coordination, and case management for relevant public health threats
	▪ Capacity for rapid mobilization and re-deployment of health workers
	▪ Initiatives to sustain health worker productivity even under stress
**Health products**	▪ Multiple options to sustain supply chain functionality
	▪ Capacity for efficient and effective management of medical supplies at all levels of the health system
**Health infrastructure**	▪ Use of available infrastructure and supplies based on need – not prescribed
	▪ Local capacity to sustain existing health facility infrastructure
**Health information**	▪ Regular health facility (at least once a year) mapping of health system assets (human resources, infrastructure, medicines)
	▪ Regular health facility mapping (at least once a year) of potential health risks in their area of responsibility
	▪ Development of a compendium of lessons learnt from responding to different shock events
	▪ Realtime capacity for process documentation and intelligence generation during shock events
	▪ Real-time surveillance of service provision and capacity to sustain essential services
**Service delivery**	▪ Districts-level stress tests to determine strengths and gaps in response capacity at least once a quarter
	▪ Clear essential health services package at facility and district level – to recognize new events
	▪ Mapping of physical, financial, and cultural barriers to access of essential services
	▪ Health facility and district-level contingency plans that define how essential services will be maintained
**Governance and coordination**	▪ Functional facility level mechanisms for communication and engagement with non-public health partners
	▪ Non-public health partners, other sectors, communities involved in planning, and monitoring processes
	▪ Appropriate decision-making authority with health facilities to facilitate early response
	▪ Clear plan for sharing staff, funds, and capacities amongst all facilities in a district
	▪ Guidance on comprehensive recovery planning for districts
**Financial management**	▪ Health facility and district awareness, including information on funds from partners for planning and resource allocation processes
	▪ Mechanism to rapidly mobilize resources through re-allocation and/or funds from partners to respond to threats

**Fig. 1:**
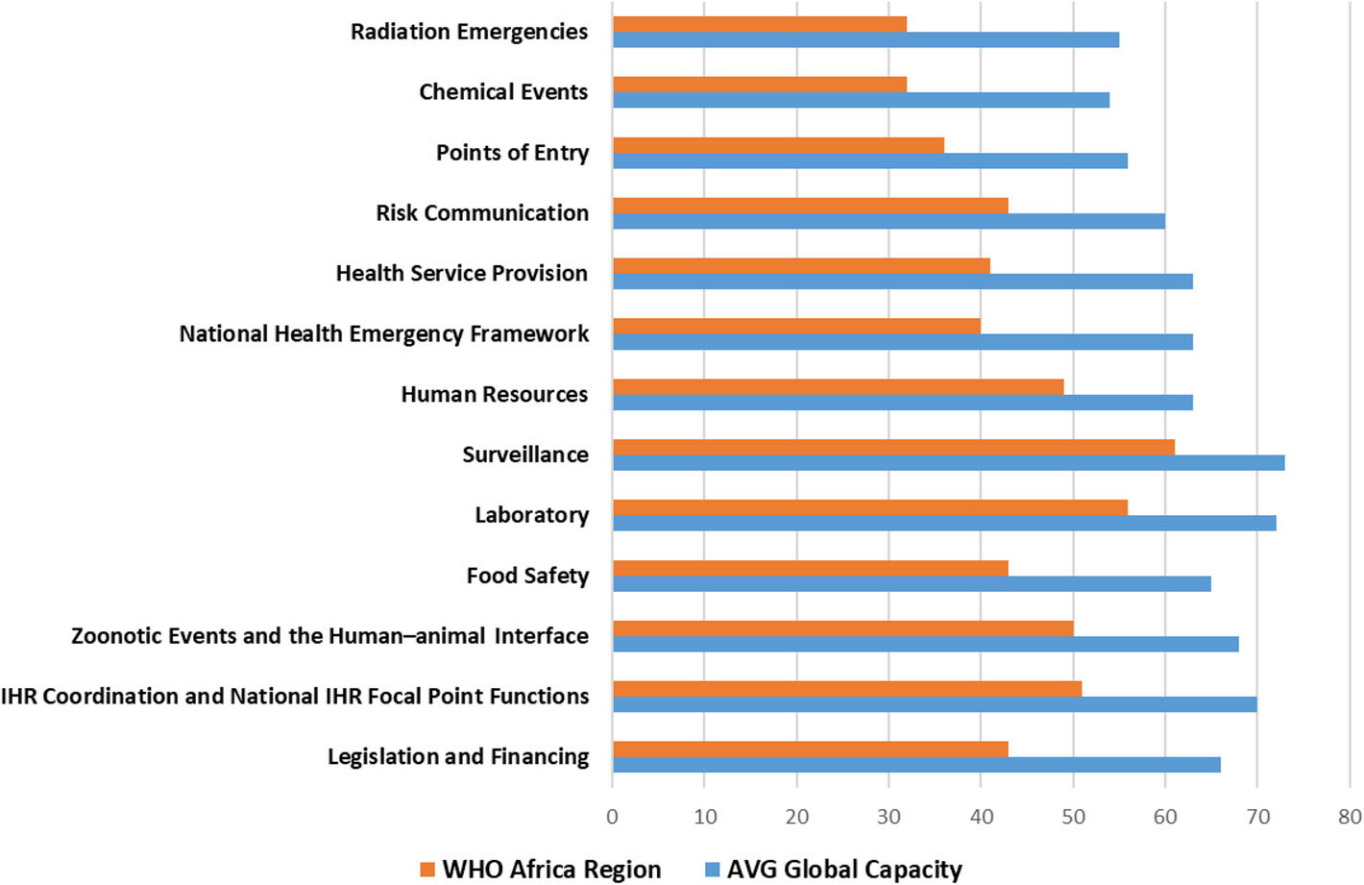
Score (out of 100) for the International Health Regulations Core Capacities, WHO Africa Region and globally [14]

Second, emergency preparedness and response, represents the other key factor of building resilient systems. Emergency preparedness and response capacity is focused on preventing, detecting, and responding to potential threats. In many countries, specific efforts to strengthen these are prioritized. However, in the African Region, the score for International Health Regulations core capacities has consistently underperformed the global average (Fig. 1). In particular, points of entry surveillance and risk communication are particularly weak, an issue that has proven problematic during the COVID-19 response. As such, focus should be afforded here to enhance emergency preparedness and response capacity.

The session also emphasized the need for enhancing health system capacity, which is focused on looking ahead and improving the functionality of our systems, as there is capacity to build the health system further. The two limiting factors include: (i) available funding for health and (ii) performance of the system (i.e. how it translates available resources to a functional system). However, financing is not crucial, as a system can perform with limited finances and a well-funded system can underperform. While lower available expenditures limit the potential to increase the overall health system capacity, this can be overcome through reducing inefficiencies in performance, as this limits the potential to increase overall health system capacity.

### Sustaining continuity of essential health services in the context of COVID-19

Maintaining essential health services is of utmost importance, as it is estimated that almost every country in the world (90%) experienced a disruption to essential health services during the COVID-19 pandemic, with larger disruptions felt in low- and middle-income countries. In fact, countries reported disruptions to about half of the 25 health services studied [15]. For example, 1.37 million children across the African Region missed their BCG vaccine and 1.32 million children aged under one year missed their first dose of the measles vaccine between January and August 2020, when compared to the same period in 2019 [15]. WHO, UNICEF and Gavi also warn that about at least 80 million children under one are at risk of diseases such as diphtheria, measles, and polio as COVID-19 disrupts routine vaccination efforts [16]. Evidently, COVID-19 has impacted individual behaviours around receiving essential health services, which in turn, negatively influences health. Between February and April 2020, compared to the same period in the previous year, maternal deaths increased by 10% in Angola, 7% in the Democratic Republic of the Congo, and 5% in Cameroon, with other indicators being similarly impacted [15].

The WHO has worked to support member states to ensure continuity of essential health services in the context of COVID-19, taking various actions, including: providing countries with strategic and operational technical guidance and supporting the development of response plans; undertaking baseline capacity assessments for maintenance of essential services to mitigate gaps; providing training to build capacity among frontline health workers and managers; and providing PPE and infection, prevention, and control capacity building to curb rising health workforce infections. For example, Rwanda and Uganda both set up COVID-19 treatment centers that are structurally separate but operationally linked to existing health facilities to minimize the risk of contamination of non-COVID-19 patients. However, despite actions undertaken to ensure the continuity of essential health services, challenges persist around: the availability of timely, regular, and robust country health management information systems; fragmented efforts by partners; inadequate ownership of the processes by some countries; and difficulty with decentralizing strategies to district levels and building capacity at the sub-national level, where the most emphasis needs to be placed, for scale-up of service continuity interventions.

With cases of resurgence and the potential for a ‘second wave’ of COVID-19, health sector directors of planning and policy are presented with an opportunity to help facilitate access to timely, regular, and complete data to aid timely detection of service disruption and adequately intervene; champion the institutionalization of health service continuity as a core component of country emergency response operations (i.e. each country should have a structure); and ensure full ownership and further health systems investments at country level, as a strategy to boost system resilience using lessons from the current COVID-19 response.

### PHC revitalization and launch of the WHO PHC operationalization framework

The WHO has revitalized PHC through undertaking extensive stakeholder consultations with various stakeholders (e.g. experts, the public, civil society, member states, and others), which has led to the development of the PHC Operational Framework. In alignment with the framework which was on the agenda of the recently concluded 73rd World Health Assembly [17], various PHC initiatives in three countries in the region were discussed: the PHC national consultation in Burundi, operationalization of the health district (Côte d’Ivoire), and the Seychelles national PHC forum; with several other countries beginning to adopt the PHC for UHC agenda.

Again, opportunity emerges for member states and directors of planning and policy, as they can: strengthen national ownership and promote strong leadership to align national and international partners on health policies based on PHC principles; mobilize additional resources (local and external) and improve management of available resources; and strengthen policy dialogues by establishing a unique coordination body that includes robust country-level coordination mechanisms.

## Discussion

This section draws on themes that emerged through active participant discussion among the participants. These themes are: (1) working multisectorally/intersectorally, (2) moving from fragmentation to integration, (3) ensuring implementation and knowledge exchange, and (4) rethinking resilience and embracing antifragility. These themes, while not proscriptively mentioned to participants, naturally emerged and align strongly with ongoing global health discussions. For example, working multisectorally through a whole-of-government approach and the need for drawing on evidence to improve health systems have been highlighted by the Director-General of the WHO in discussions of PHC [4].

### Working multisectorally/intersectorally

A widely raised theme was the need to work multisectorally/intersectorally. Whether generally or in terms of specific approaches, such as the Health in All Policies approach, the need to work multisectorally/intersectorally was brought up by numerous participants, reinforcing the identification of multisectoral policy and action as a core pillar of PHC [4]. There were various perspectives as to why this crucial. First, there was an expressed understanding of the existence of a humanitarian and development divide, which lends itself to different approaches, ways of thinking, and practical challenges such as the coordination of interventions. Guidance on how to proceed to bridge this divide was requested from the WHO. Second, there was an understanding that much of the health issues that ministries of health deal with are ‘spillovers’ from other sectors (e.g. cholera needs to be addressed by water and sanitation, road traffic accidents to be addressed by transportation). Particularly with returning cholera outbreaks, the need to work across sectors to address these various health issues at the ‘root’, becomes increasingly important. Third, the need to work multisectorally with attention paid to working with key actors who have power (e.g. Ministry of Finance who is responsible for allocating the budget) was raised. And forth, the need to work with politicians was raised due to the political nature of health and need to gain legislative support, which is an important component of governance. Similarly, there is an opportunity to move towards developing legislation that supports health. For example, facilitating coordination, partnership, integration, healthcare financing, monitoring & evaluation, and addressing corruption and upstream population cultural and societal practices.

Overall, as one participant indicated, there is a need for *joint* planning with *joint* implementation and monitoring and evaluation on *jointly* determined priorities. This was identified as needing to be integrated over the mid- to long-term. Through working across sectors and reducing waste, efficiency may be increased, leading to improved value-for-money.

### Moving from fragmentation to integration

The second theme emerged around needing to move from fragmentation to integration. In many countries, the national emergency program operates within its own structure and allocated resources and sometimes in parallel with the health system. This is problematic as the fragmentation of health programs limits the capacity for resilience. As a result, the need to think about how routine and emergency health services can be brought together into one structure was raised. For example, beginning the process of integrating National Action Plans for Health Security with National Health Sector Strategic Plans, expanding health sector monitoring and evaluation plans to incorporate indicators on system resilience and health security, and handling quality of care in a holistic manner (e.g. quality of care network).

Similarly, drawing on the benefits of integration, opportunities emerge. For example, in Chad, integrating flagship activities within the framework of funding, which allocates specific resources to vulnerable persons as per national law implemented in 2019, has allowed for a focus on select groups, including: workers, those in the informal sector, and vulnerable persons.

### Ensuring implementation and knowledge exchange

The third theme was raised around the difficulty in successfully moving a plan forward from conceptualization to implementation. Participants raised questions around how plans can be translated into resource needs and then appropriately generating these resources in a coordinated and integrated manner. Rightfully drawing attention to the link between limited or non-existent capacity and lack of implementation. To overcome this, a suggestion was put forward to cost all plans and align with national budgets to allow for smoother implementation.

Alongside these discussions, points around the availability of data and knowledge exchange were raised. This discussion is complementary to the above, as availability of data and exchange of knowledge will allow for enhanced implementation. Participants indicated that they face problems with a lack of or limited data and that when provided, it arrives late. For example, one participant requested information on proven interventions for reducing maternal and under-five mortality and another on best practices for allocating finances at the country-level. As such, opportunity emerges for infrastructure that allows for information transmission in a timely manner.

Through this discussion, various suggestions and recommendations were put forward to the WHO. First, it was suggested that the WHO develop a ‘marketplace’ or compendium of lessons learned hosted at AFRO for countries to look to and learn from country experiences. Second, WHO can offer support for standardizing and elaborating health interventions at various levels of the service delivery system, to enable streamlining. Third, to develop a guideline of best practices for allocation of finances at the country-level (with a comparative table of all countries). Fourth, development of infrastructure that allows for information transmission in a timely manner. And fifth, establishing peer-to-peer or exchange opportunities within the region.

### Rethinking resilience and embracing antifragility

The last theme that emerged through the participant discussion was around the need to emphasize the importance of resilience and embrace antifragility. Resilience entails the capacity to sustain essential services provision, even in the context of shock events, while antifragility moves beyond resilience.

Typically, resilience is considered in the context of individuals who are in an immediate need of services. However, we should also consider building the inherent resilience capacity of systems to equally provide services for healthy people, such as through health promotion and disease prevention. Focusing on these interventions, will reduce the burden on health facilities.

In order to rethink resilience and embrace antifragility, learning once a shock has been felt should be emphasized. In other words, increased attention should be placed on how a system can learn from a shock and improve to better respond in the future. Guinea shared their experience drawing on lessons learned to curb the negative impacts of COVID-19 which demonstrates the importance of this endeavor. In Guinea, following the shock caused by the 2014–16 EVD epidemic, a plan was put in place to train epidemiologists on the ground. Because these structures were implemented prior to the onset of COVID-19, challenges, such with infrastructure, equipment, and other resources, were not faced. Evidently, having these structures in place prior to COVID-19 afforded various benefits (e.g. reduced expenditures). However, with COVID-19, additional lessons learned have emerged, such as the need for real-time mortality and morbidity data, allowing for additional adaptation and growth.

## Conclusion

In closing, it was emphasized that “a health system by design should be resilient”. In line with this view, consideration should be afforded to interrogate how the health system has been constructed (e.g. is it robust?). The way health systems are designed matters in terms of the ability to adapt, change, anticipate, and therefore, put into place contingency plans that can be put into action when need arises.

Naturally, COVID-19 has afforded lessons in policy development, including ensuring policy solutions are strategic and forward-thinking, as opposed to short-sighted solutions, and the need to focus on equity, effectiveness, and efficiency, where relevant [18]. Attention should be paid to moving the health system from one of responding to the crisis at-hand to being more proactive and planning upstream, leaving no ne behind. “Pre-existing policy problems not adequately addressed exacerbate the cost of crises (including deaths) and make policy responses more difficult” [18]. While discussed more broadly (e.g. in terms of social safety nets), the conclusion is highly applicable here. There is an opportunity to properly address UHC and PHC, as it will only become more difficult in the future with additional crisis.

**Fig. 2:**
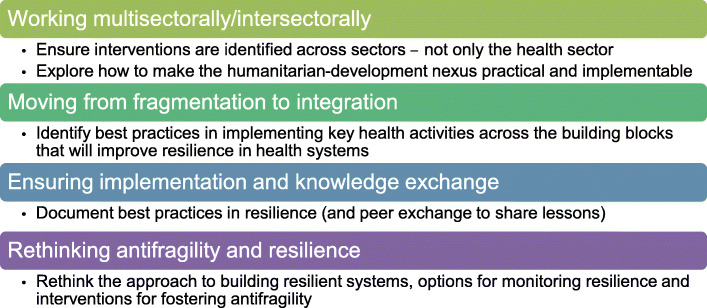
Recommendations emergent from participant discussion, organized by theme

Efforts should be made to ensure policy solutions devised draw on lessons learned and are durable. At present, this is evident when considering the importance of multisectoral action that emphasizes whole-of-government and whole-of-society approaches, as these remain neglected and underfunded [4]. As discussed at the Regional Forum, there is an urgent need to sustain the continuity of essential health services in the context of COVID-19, which can be greatly strengthened by scaling-up the PHC approach. Commitment to engaging in critical discussions, particularly around emergent themes from this Regional Forum, to further action in a deliberate manner must be secured. From the key themes, recommendations have emerged for the consideration of the WHO, which are detailed in Fig. 2.

Through hosting this Regional Forum, the WHO facilitated collaborations that promote knowledge translation and exchange to promote better policymaking. The forum reiterated the commitment of the WHO Regional Office to improving the health and well-being of all, through its dedicated support to member states on this road to UHC attainment and the overall 2030 agenda.

## Data Availability

Not applicable.

## Abbreviations

PHC: Primary Health Care
SDGs: Sustainable Development Goals
UHC: Universal Health Coverage
WHO: World Health Organization
